# Prediction of Tribological Properties of UHMWPE/SiC Polymer Composites Using Machine Learning Techniques

**DOI:** 10.3390/polym15204057

**Published:** 2023-10-11

**Authors:** Abdul Jawad Mohammed, Anwaruddin Siddiqui Mohammed, Abdul Samad Mohammed

**Affiliations:** 1 Department of Information and Computer Science, King Fahd University of Petroleum and Minerals, Dhahran 31261, Saudi Arabia; jawad.isg@gmail.com; 2 Mechanical Engineering Department, Wichita State University, Wichita, KS 67260, USA; axmohammed15@shockers.wichita.edu; 3 Department of Mechanical Engineering, King Fahd University of Petroleum and Minerals, Dhahran 31261, Saudi Arabia; 4 Interdisciplinary Research Center for Advanced Materials, King Fahd University of Petroleum and Minerals, Dhahran 31261, Saudi Arabia

**Keywords:** machine learning, UHMWPE, silicon carbide, friction, wear rate, triboinformatics

## Abstract

Polymer composites are a class of material that are gaining a lot of attention in demanding tribological applications due to the ability of manipulating their performance by changing various factors, such as processing parameters, types of fillers, and operational parameters. Hence, a number of samples under different conditions need to be repeatedly produced and tested in order to satisfy the requirements of an application. However, with the advent of a new field of triboinformatics, which is a scientific discipline involving computer technology to collect, store, analyze, and evaluate tribological properties, we presently have access to a variety of high-end tools, such as various machine learning (ML) techniques, which can significantly aid in efficiently gauging the polymer’s characteristics without the need to invest time and money in a physical experimentation. The development of an accurate model specifically for predicting the properties of the composite would not only cheapen the process of product testing, but also bolster the production rates of a very strong polymer combination. Hence, in the current study, the performance of five different machine learning (ML) techniques is evaluated for accurately predicting the tribological properties of ultrahigh molecular-weight polyethylene (UHMWPE) polymer composites reinforced with silicon carbide (SiC) nanoparticles. Three input parameters, namely, the applied pressure, holding time, and the concentration of SiCs, are considered with the specific wear rate (SWR) and coefficient of friction (COF) as the two output parameters. The five techniques used are support vector machines (SVMs), decision trees (DTs), random forests (RFs), k-nearest neighbors (KNNs), and artificial neural networks (ANNs). Three evaluation statistical metrics, namely, the coefficient of determination (R^2^-value), mean absolute error (MAE), and root mean square error (RMSE), are used to evaluate and compare the performances of the different ML techniques. Based upon the experimental dataset, the SVM technique was observed to yield the lowest error rates—with the RMSE being 2.09 × 10^−4^ and MAE being 2 × 10^−4^ for COF and for SWR, an RMSE of 2 × 10^−4^ and MAE of 1.6 × 10^−4^ were obtained—and highest R^2^-values of 0.9999 for COF and 0.9998 for SWR. The observed performance metrics shows the SVM as the most reliable technique in predicting the tribological properties—with an accuracy of 99.99% for COF and 99.98% for SWR—of the polymer composites.

## 1. Introduction

The applications of polymers for bearings have been increasing due to their low friction, low cost, and ease of manufacturing compared to metallic bearings. However, polymers suffer from medium-to-higher wear rates, especially at elevated temperatures. Hence, in order to improve their wear resistance and overcome other shortcomings, polymers can be reinforced with small loads of other synthetic compounds called fillers. Extensive research has been conducted for various types of polymers and reinforcement compounds to create combinations with optimal mechanical and tribological properties. Polytetrafluoroethylene (PTFE), polyetherether ether ketone (PEEK), and ultrahigh molecular polyethylene are some of the polymers that have been comprehensively investigated as a parent matrix because of their excellent tribological properties. Fillers, such as carbon naotubes (CNTs), graphene, graphite, nanoclay, alumina, silicon carbide (SiC), etc., have been used to reinforce these polymers to further improve their properties so that they can be used in demanding applications. To obtain the best properties and performances of the composites, different factors have to be optimized meticulously. Factors, such as the number of fillers, processing parameters (pressure, time, and temperature), and operational parameters (load, speed, and temperature), to name a few, play a very important role in determining the characteristics of the developed composites. Hence, different proportions of fillers need to be repeatedly produced and tested under varying conditions in order to satisfy the requirements of the respective application. However, with the advent of a new field of triboinformatics, which is a scientific discipline involving computer technology to collect, store, analyze, and evaluate tribological properties, we presently have access to a variety of high-end tools, such as various machine learning (ML) techniques, which can significantly aid in efficiently gauging the polymer’s characteristics without the need to invest time and money in a physical experimentation. The development of an accurate model specifically for predicting the properties of the composite would not only cheapen the process of product testing, but also bolster the production rates of a very strong polymer combination.

Various researchers have applied this powerful tool of machine learning in a variety of fields, such as biochemistry [1], biomedicine [2,3], and material science [4,5], to name a few. The applications of machine learning in predicting the material characteristics of polymers has also been looked into by numerous researchers. Khanan et al. [6] utilized artificial neural networks trained on experimental data and developed a model to approximate the thermal and physical properties of graphene nanocomposite samples, yielding strong correlation values of around 0.98–0.99 for predictions on five unseen samples. Vinodh et al. [7] also utilized artificial neural network models to predict the tribological features for UHMWPE composites reinforced with graphene and carbon fiber. A dataset of 624 points was collected from published sources to be used for training the models. The logistic regression algorithm, a popular supervised machine learning technique, was used by Kiakkojouri A et al. [8] to obtain a fault classification of rolling element bearing. Other techniques, such as support vector machines (SVMs), were employed by Nazari and Sanjayan [9] in combination with a number of optimization algorithms to predict the compressive strength of geopolymer paste. SVMs were the subject of another study conducted by Zhang and Wang [10], who utilized them to analyze the properties of concrete-reinforced polymers.

There have also been several studies utilizing different machine learning techniques to compare each of them and predict different tribological properties [11,12,13]. The coefficient of friction of SU-8, an epoxy polymer, was predicted utilizing the input parameters as number of cycles and the filler-type used, utilizing various algorithms, such as ANN, SVM, RF, and CART, and it was found that for the set of experimental data used to train the different algorithms, ANN was observed to provide best performance metrics [14]. A similar study was also conducted where different machine learning techniques were used to predict the wear from pin-on-disc-type experiments by varying different operating parameters [15]. An attempt is made in the present study to mimic the trend of comparing and predicting the tribological performances utilizing different machine learning techniques from the experimental data. Hence, the main focus of the current study is to use the experimental dataset of Aliyu et al. [16] and implement five different ML techniques, namely, support vector machines (SVMs), decision trees (DTs), random forests (RFs), k-nearest neighbors (KNN), and artificial neural networks (ANN), to compare their performances in terms of accurately predicting the tribological properties of the composites.

The primary objectives of the current research are as follows:

The expansion of the relatively small amount of data produced by [16] for the prediction of the tribological characteristics of the UHMWPE/SiC composites under even more fine-tuned scenarios that would be otherwise difficult to realize physically.

Implementation and fine-tuning of five machine learning algorithms to create prediction models for UHMWPE properties.

Comparison and regression analysis of the machine learning models to determine the best-performing model for the selected dataset.

## 2. Experimental Procedure

The study conducted by Aliyu et al. [16], which was the focus of our research, applied the Taguchi methodology to explore the reinforcement of ultrahigh molecular-weight polyethylene (UHMWPE) with varying loads of silicon carbide nanoparticles while observing the friction and wear rates of the resulting combinations. The experiment consisted of 9 trials under 3 independent variables: silicon carbide loading (as a percent of the polymer’s total weight), consolidation pressure, and holding time. The two output variables measured were the specific wear rate (SWR) and coefficient of friction (COF).

The procedure followed by [16] for preparing the powders, bulk composites, and conducting the wear tests is described here for a better understanding. Each sample’s required loading of SiC was first added to 50 mL of ethanol and then subjected to a 7 min sonication process. The mixture was then combined with the proper amount of UHMWPE, bringing the total mass to 10 g, and sonicated for 14 min. Then, a magnetic stirrer was used to agitate the mixture for 30 min at 600 rpm. After draining the ethanol, the powder was dried for 24 h at 60 °C in an oven. Hot pressing was employed to fabricate the bulk nanocomposites using the produced powders. For each sample, the consolidation pressure and holding time in accordance with L9 OA of the Taguchi design (Table 1 and Table 2) were chosen. All samples were consolidated at a temperature of 175 °C, followed by a cooling time of 20 min.

The samples’ wear rates and coefficients of friction were evaluated using a Bruker UMT-3 Tribometer. In order to imitate plain sliding bearing and guarantee that the contact pressure remained constant during the test, a pin-on-disk wear-test configuration was used. The counterface was a 6.35 mm silver steel pin with a surface roughness of 0.43 ± 0.04 µm and a contact end diameter of 2.6 mm as a result of chamfering. Prior to the wear test, the pin was hardened by heat treatment to around 58 HRC. Wear tests were conducted at a linear speed of 0.5 m/s, a load of 64 N, with an equivalent contact pressure of 12 MPa.

Each of the tests was run for a sliding distance of 500 m at room temperature and at a relative humidity of between 48 to 55%. After each wear test, the wear-track cross-sectional area was measured using a GTK-A optical profilometer from Bruker Co. The average track circumference was multiplied by the wear track’s area to obtain the wear volume, which was then used to calculate the SWR in accordance with Equation (1).

$$w=\frac{V}{F\times D}$$

where *w* is the SWR in mm^3^ (Nm)^−1^, *F* is the normal force, and *D* is the linear sliding-distance traveled.

In the current study, we explored the possibility of accurately predicting the tribological behavior of these developed polymer composites for a range of unobserved scenarios that were within the bounds of the experimented values by using the prowess of machine learning techniques. Table 1 and Table 2 present the experimental data in terms of the input variables that they use and the corresponding measured output variables from the experiments.

### 2.1. Methodology

The general research methodology adopted in the current study is listed below. To develop a model that accurately predicted the tribological properties of UHMWPE and silicon carbide nanocomposites under varying conditions, the experimental data yielded by [16] were used as the basis for our machine learning-based research.

The methodology was as follows:

- Nine data points from the suggested study [16] were collected. To train the models, the SiC loading size, consolidation pressure, and holding time were used as features to determine the coefficient of friction (COF) and specific wear rate (SWR) of the resulting polymer composite combination.
- Using the Mathematica language, two interpolation functions that best represented the correlation between the experimental independent and dependent variables were developed for the SWR and COF from the data points of [16], as shown in Equations (1) and (2), where the variables *x*, *y*, and *z* represent SiC loading, consolidation pressure, and holding time, respectively:

(1) $$\begin{array}{ll} COF= & 0.189111+(y*(0.00280556-(0.000106481*y)+(0.000244444*z)))+(x\\ & \;\;\;\;\;\;*(0.00788889-(0.000814815*x)-(0.000425926*y)+(0.000555556\\ & \;\;\;\;\;\;*z)))-(0.00673333*z) \end{array}$$

(2) $$\begin{array}{ll} SWR= & 5.55556e-7+(x*(-1.2963e-6+(7.40741e-8*x)+(1.48148e-7*y)\\ & \;\;\;\;\;\;-(1.77778e-7*z)))+(y*(4.90741e-7-(1.38889e-8*y)\\ & \;\;\;\;\;\;-(6.66667e-8*z))+(2.11111e-6*z)) \end{array}$$

**Figure 1:**
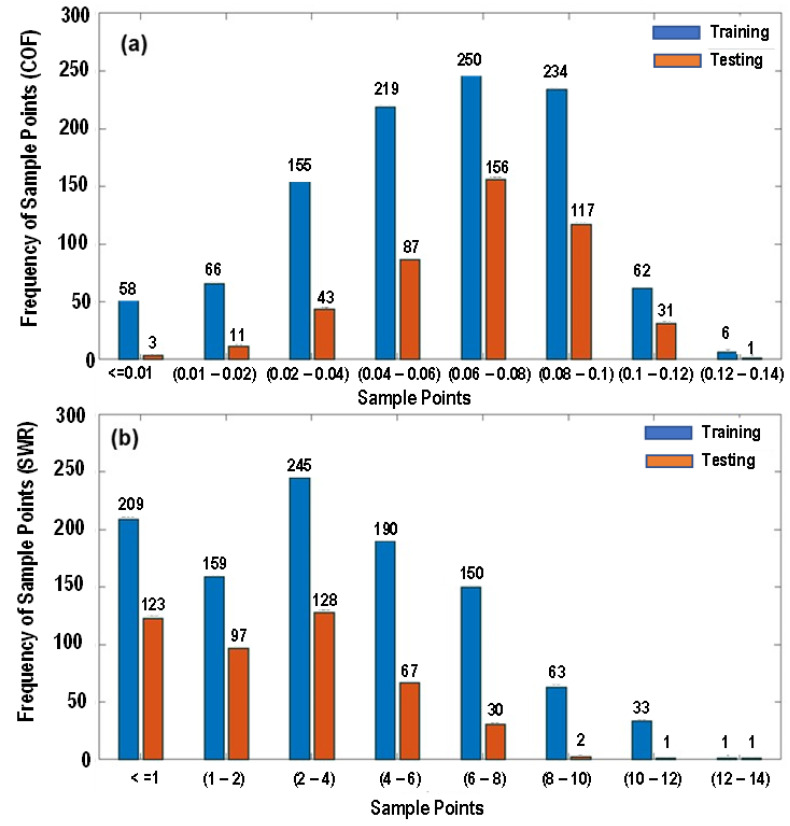
Frequency of dataset, (**a**) coefficient of friction, and (**b**) specific wear rate.

- The interpolation functions were then used to generate a larger dataset consisting of 1499 data points within the bounds of the original nine points.
- The five appropriate machine learning techniques selected were decision trees, random forests, k-nearest neighbors, support vector machine, and artificial neural network.
- The dataset was partitioned into 2 components as 70% of the samples were used to train the models, while the remaining 30% were used as an unseen test set for models to predict values. Figure 1a and Figure 1b show the frequency of the training and the testing dataset for the COF and SWR, respectively.
- Using Python, each technique was subjected to a grid search, or the repeated training of the algorithm on a set of data while varying the hyperparameters for each iteration, in order to find the optimal settings that produced the least error rates for each target variable (CoF and SWR). The data points were also rescaled depending on the method.
- The model performances were then compared with each other through regression and error analyses. In order to observe the best performance, the primary metrics used for the comparison consisted of the following:
  (a) Root mean squared error (RMSE): a measure of how far off the predictions are from the true values of the test data points. A lower RMSE closer to 0 was indicative of a better performance. The distance measure is calculated as shown in Equation (1), where $x_{i}$ is the predicted value and *x* is the true value of each sample.
    (3) $$RMSE=\sqrt{\frac{1}{n}\sum_{i=1}^{n}{\left(x_{i}-x\right)}^{2}}$$
  (b) Mean absolute error (MAE): similar to the RMSE, the MAE is also used as a metric to observe the gap in errors between the predictions and true values of the samples. The value is calculated according to Equation (2), where $x_{i}$ is the predicted value and *x* is the true value of each sample.
    (4) $$MAE=\frac{1}{n}\sum_{i=1}^{n}\left|x_{i}-x\right|$$
  (c) Coefficient of determination (R^2^-score): also known as the R-squared value, it is a measure of the correlation between the dependent and independent variables. Ranging from 0 to 1.0 (or 0 to 100%), a higher value is indicative of a better regression fit. The coefficient of determination is calculated, as shown in Equation (3), where ${\hat{x}}_{i}$ is the predicted value of the sample, $\bar{x}$ is the mean of all sample points, and $x_{i}$ is the true value.
    (5) $$R^{2}=\frac{\sum {\left({\hat{x}}_{i}-\bar{x}\right)}^{2}}{{\sum \left(x_{i}-\bar{x}\right)}^{2}}$$

### 2.2. An Overview of Machine Learning

In a world driven by endless streams of information and numerous variables that can become increasingly difficult to manage, machine learning can help in automating this process through utilizing the known data for predicting various problem-dependent outcomes in a similar manner to human beings. A machine learning model is usually first given a set of training data in order to familiarize itself with the relations and rules between the different aspects of the information that may affect the predicted outcome, or the features. The model is then made to predict the values of unseen test data, based on what it already ‘knows’. With numerous scenarios, each consisting of unique types of features, the user can tune the model’s settings, also known as hyperparameters, to fit the problem at hand. Most tasks involve either predicting the class of each data point (classification) or finding the line of best fit for a set of data values (regression). Machine learning algorithms usually consist of two types, namely, supervised learning and unsupervised learning. A type of learning where labeled data are fed through the algorithm so that the model can first train for known information before being made to predict the labels/values of unseen data based on its knowledge is termed supervised learning. A type of learning where unlabeled data are fed through the model for it to deduce information patterns by itself, without supervision, is termed unsupervised learning. It is commonly used for grouping data points according to the similarities and extracting rules. Machine learning techniques have been used by tribology experts in order to relate the complex non-linear tribological/mechanical relationship involving various parameters [17,18,19]. Some of the commonly used machine learning algorithms used in the literature for predicting tribological non-linear complex relations include artificial neural networks, support vector machine, classification and regression tree (CART), random forest, k-nearest neighbors, etc. [11,20,21].

### 2.3. Machine Learning Techniques Implemented in the Current Study

In order to determine an accurate regression model that can accurately predict tribological properties, five machine learning algorithms were implemented and their performances were compared against each other. The five different machine learning algorithms were decision trees, random forests, k-nearest neighbor, support vector machine, and artificial neural networks; their structures and the hyperparameters we tuned are explored in this section.

**Figure 2:**
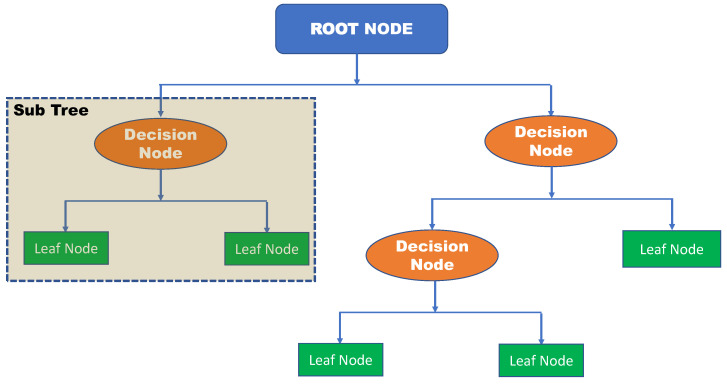
Structure of a typical decision tree.

Decision trees are a form of supervised machine learning consisting of a set of nodes and leaves. Within each node, a feature is chosen according to certain hyperparameters pertaining to the data’s variance, correlation, etc., for deciding how it should be divided to create child nodes that, in turn, divide the data’s subsets in a similar manner. As shown in Figure 2, a tree contains branches of decision nodes that keep dividing the data until no more separations can occur, concluding with a leaf node, and the optimization can be obtained by varying the depth and number of leaves per node [22,23]. Decision trees are commonly utilized for their easy interpretation [24] and not needing input data to be normalized or rescaled. However, they are also significantly unstable as one change in a hyperparameter can produce drastically different results.

For our experiments, most of the decision tree algorithm’s hyperparameters were left unchanged as their manipulation was observed to increase the error rates when predicting the test data. However, the maximum depth, or the limit of how much a tree could sub-divide the data, was fine-tuned in order to not cause the model to excessively ‘memorize’ the training data (also known as overfitting).

**Figure 3:**
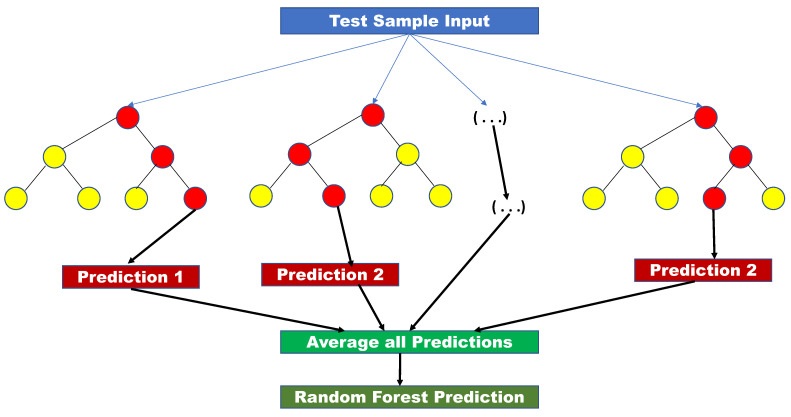
Random forest structure of three decision trees.

Random forests are combinations of multiple decision trees (see Figure 3) that each produce a value or decided label for the given data. The outputs are then averaged or voted upon before a final decision is made for the output. Being an ensemble of multiple trees, random forests generally produce more accurate metrics when compared to their single-tree counterparts, taking extra consideration to the importance of each feature into account [24,25,26]. However, they share similar levels of instability and hyperparameter sensitivity with decision trees. Certain hyperparameters were manipulated during the training, including the minimum split, minimum leaf sample, and impurity decrease threshold, which was the measure of data uniformity within a node, which indeed was the basic idea that a random forest or decision tree classifier was based on to achieve a uniformity of data within subsequent nodes.

**Figure 4:**
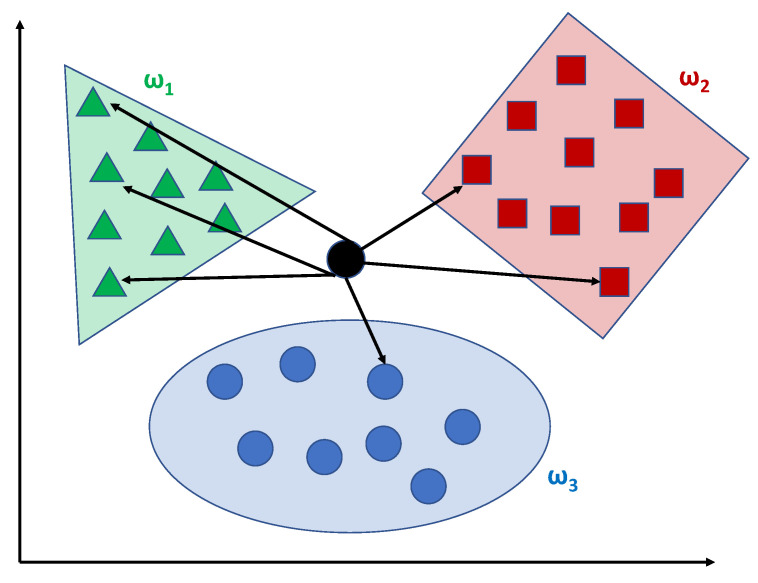
K-nearest neighbors.

KNNs are a type of supervised learning that involves the grouping of data points based on proximity or distance. It primarily functions on the concept of similarity (Figure 4), or that data points sharing commonalities tend to be ‘closer’ to each other. KNNs do not require any training time since the ‘learning’ is performed during the distance calculation between each unseen test point and every one of the training points in the dataset to determine the closest proximity. Due to this, more data can be efficiently added to the model, making it highly scalable. Moreover, KNNs usually perform better with a lower number of features, regressing in performance with more dimensions [10]. Unlike decision trees and random forests, however, KNNs require the data to be normalized beforehand to prevent a disparity in the value ranges. Moreover, noise and outlier data can hinder the model’s performance due to its high sensitivity [27,28]. The hyperparameters varied during the implementation of the algorithm were the number of neighbors, which set the number of neighboring points to consider before averaging; weights, which attempted to design the impact of the neighboring training samples in distance calculations; and the distance computation, the type of method used for distance calculations, such as Manhattan or Euclidean distances.

**Figure 5:**
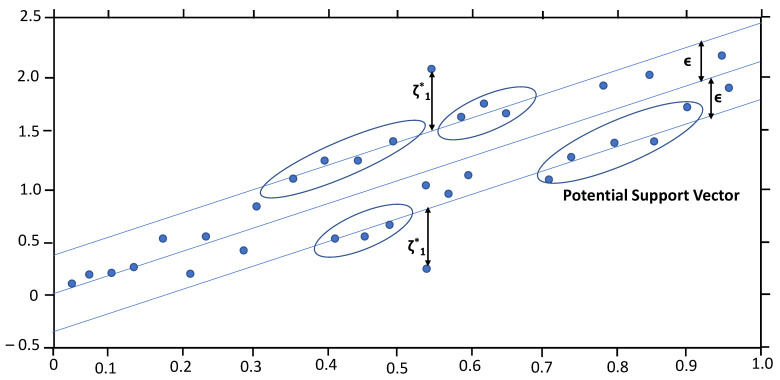
SVM margin.

SVMs are supervised learning algorithms that focus on finding the ‘perfect’ margin or margins that minimize the errors between predicted and actual values. As shown in Figure 5, the algorithm focuses on finding the margin with the narrowest width, with the points on the boundary line (known as support vectors) being used for optimizing the margin. Support vector machines are robust against outliers and biases in the data, converging (finding an optimal solution) faster than most other machine learning techniques. Moreover, the versatility of their hyperparameters provides for a range of customizable options for dealing with various kinds of problems [29,30]. Variation in the different hyperparameters, such as kernel function, gamma, C parameter, and epsilon, were created to check for the hyperparameters’ optimal limits.

ANNs perform feature extractions on training data through multiple layers, each with a designated purpose. With each layer being an assembly of neurons that compute information importance (or weights) and connect to those of the adjacent layers, neural networks are arguably the closest in resemblance to the human brain among other machine learning techniques. There have been many studies in the literature utilizing ANNs to predict different parameters in the field of material science [31,32].

**Figure 6:**
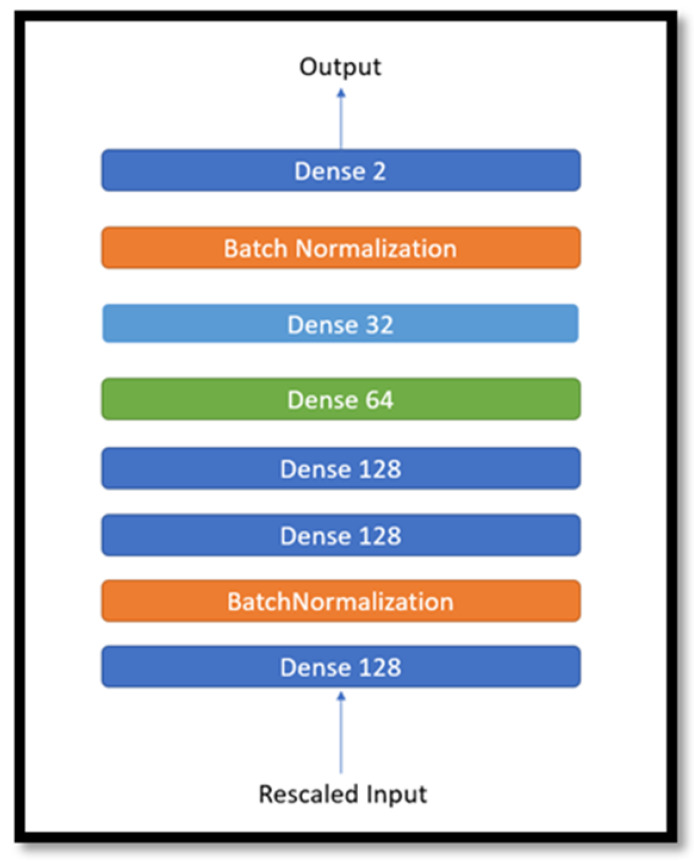
ANN model finalized during the experiments.

Figure 6 shows the developed ANN model structure, trained for 100 epochs or training loops, which was found to produce the least error rates on our dataset during the experimental procedure; the numbers represent the number of neurons. It primarily consists of two types of layers, which are dense and batch normalization layers. Dense layers are groups of interconnected neurons that feed outputs from previous layers onto the succeeding layer. They form an important framework of the model as they connect other layers, in addition to updating the trainable aspects of the network, such as weights and biases. The batch normalization layers act as regularizers, normalizing input information and keeping the standard deviation close to 1, in addition to not ensuring only the perfect memorization of the training data but also understanding the impact of the features on the Cof and SWR. In addition to controlling the dense and batch normalization layers, some other hyperparameters were also varied to check for the optimal performance of the network that included the optimizer/activation function, learning rate, epochs, and validation split.

## 3. Results and Analysis

### 3.1. Performance Analysis

The experimental phase of the research concluded with all five machine learning techniques yielding R-squared values within the 0.95–0.99 range for both the coefficient of friction and specific wear rate (shown in Table 3) when predicting the test set.

The metrics achieved by each model and the best hyperparameters observed are briefly elaborated upon in the following sections.

#### 3.1.1. Decision Tree

**Figure 7:**
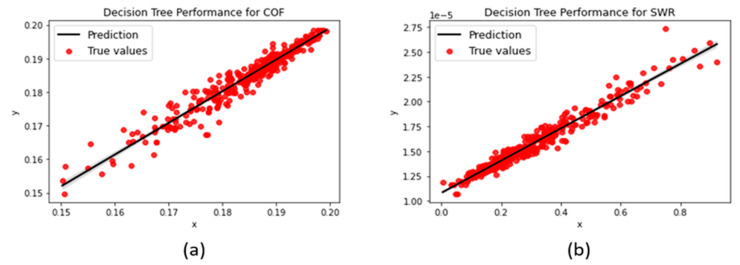
Decision tree regression performances for (**a**) COF and (**b**) SWR predictions.

Due to being sensitive to adjustments in the hyperparameters, the decision tree model was one of the more difficult algorithms to train. Changes in the model settings pertaining to the minimum samples needed in each node or leaf, the type of splitting, etc., produced negative R^2^-scores and relatively high MAE/RMSEs that were closer to 0.1. Moreover, the absence of a depth limit caused the model to excessively conform to the training data and hindered its ability to predict unseen data. Limiting the maximum depth to 50 while reflecting the default settings by keeping minimum node samples at 2 and leaf samples at 1, we achieved more favorable error metrics (as shown in Figure 7) with the model.

#### 3.1.2. Random Forest

**Figure 8:**
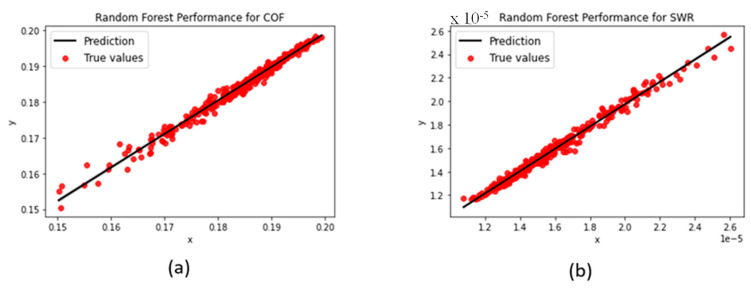
Random forest regression performances for (**a**) COF and (**b**) SWR.

While sharing similar sensitivity levels to hyperparameter turning with the decision tree, the random forest model presented greater stability, allowing for different aspects to be adjusted without drastic fluctuations in its performance. The lack of a maximum depth limit did not seem to deter its prediction ability, as it was still able to achieve high R^2^-scores and a very strong fit for both COF and SWR predictions (shown in Figure 8).

#### 3.1.3. K-Nearest Neighbors

**Figure 9:**
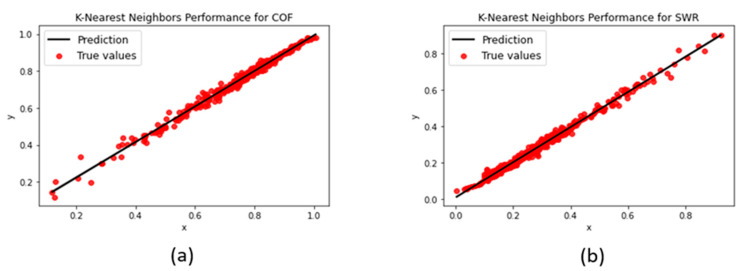
KNN regression performances for (**a**) COF and (**b**) SWR.

The KNN model yielded the most consistent error metrics out of all the models during the grid search, achieving R^2^-scores within the 0.95–0.99 range and MAE/RMSEs from 0.013 to 0.016. A possible explanation for its astounding performance and exceptional prediction fit (as shown in Figure 9) can be that the data possessed only three input features, aligning with the strengths of the model in lower dimensions.

#### 3.1.4. Support Vector Machine

**Figure 10:**
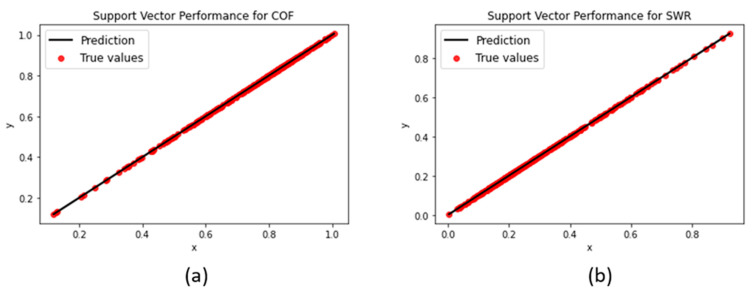
SVM regression performances for (**a**) COF and (**b**) SWR.

The support vector machine yielded the highest R^2^-score out of all models for both COF and SWR when using a gamma value of 0.25, C parameter of 100, and epsilon of 0.00005. A high C parameter and low epsilon value led to the model narrowing its margin as much as possible in order to attain an optimal prediction fit (shown in Figure 10).

#### 3.1.5. Artificial Neural Network

**Figure 11:**
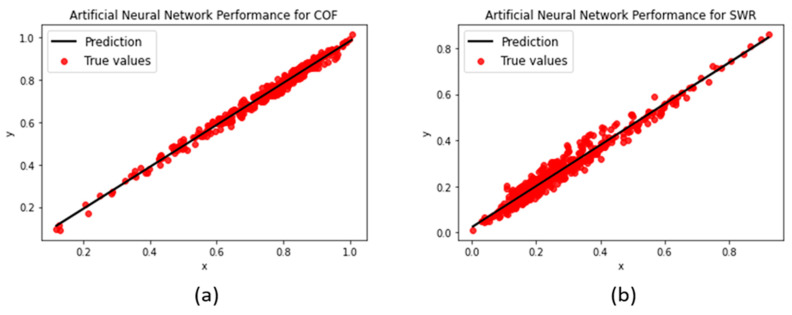
ANN regression performances for (**a**) COF and (**b**) SWR.

The numerous modifiable components of the ANN model cemented it as the most complex of the machine learning techniques to experiment with. The types and number of layers, their order within the structure, the number of neurons to initialize, the optimizer algorithm, etc., all had to be tuned and run through a number of epochs, before being retested. While the models were initially trained for 50 epochs with 64 neurons in each layer, an increase of 16.8% in the R^2^-score (0.83 to 0.97) for COF was observed when trained for 100 epochs. Doubling the number of neurons in each dense layer was observed to increase the R^2^-scores by 6.5 to 7% for the specific wear rate and 19.7% for the coefficient of friction, possibly due to the improved processing of information from more neurons (shown in Figure 11).

## 4. Future Extension

The realm of machine learning is bottomless and dynamic. Even within the five machine learning techniques that were implemented to develop an accurate prediction model, there were unseen potential values for the various hyperparameters and how they synergized with one another. We also plan to explore the vast field of neural networks and how our current models can be further bolstered to achieve lower error rates and higher R^2^-scores, not just for UHMWPE/SiC composites but for other material types as well. Moreover, working on random forest models manifested the idea of further studying ensemble models for our tribological problem.

## 5. Conclusions

Among the techniques implemented for predicting the COFs and SWRs of the UHMWPE-SiC nanocomposite samples, the optimizing and marginalizing nature of the support vector machine gave it the edge in terms of producing the highest R^2^-score (0.9999) along with an almost perfect prediction fit, followed by the K-nearest neighbor model (R^2^ value—0.98891). Moreover, the random forest’s multi-decision tree ensemble structure proved to be formidable, barely trailing behind in terms of the R^2^-score (0.9827), while also achieving the lowest RMSE (3.1 × 10^−7^) and MAE (2.1 × 10^−7^) scored for the specific wear rate. The ANN was outshone by the other models in all of the error metrics by at least 28%, likely due to it needing significantly more data for training to compensate for its higher complexity.

In conclusion, the result metrics showcase the strengths of traditional machine learning algorithms for performing regression analyses in triboinformatic scenarios incorporating a relatively small amount of data. While often overlooked at present, in favor of deep learning architectures, the more intuitive and lightweight nature of machine learning models can prove to be beneficial for smaller-scale experiments.

Despite providing a glimpse of the potential of harnessing the power of machine learning for material analyses, this research represents only a microcosm of the infinite possibilities that can result from this amalgamation of fields of study.

## Figures and Tables

**Table 1:** COF results obtained from the 9 trials conducted by Aliyu et al. [16].

Trial No.	SiC Loading (wt%)	Consolidation Pressure (MPa)	Holding Time (min)	COF
1	1	10	10	0.172
2	1	16	15	0.173
3	1	22	20	0.181
4	4	10	15	0.177
5	4	16	20	0.186
6	4	22	10	0.189
7	7	10	20	0.184
8	7	16	10	0.185
9	7	22	15	0.187

**Table 2:** SWR results obtained from the 9 trials conducted by Aliyu et al. [16].

Trial No.	SiC Loading (wt%)	Consolidation Pressure (MPa)	Holding Time (min)	SWR × 10^−5^ (mm^3^/Nm)
1	1	10	10	1.65
2	1	16	15	1.89
3	1	22	20	1.55
4	4	10	15	1.68
5	4	16	20	1.69
6	4	22	10	1.35
7	7	10	20	1.29
8	7	16	10	1.40
9	7	22	15	1.26

**Table 3:** Summary of the error metrics obtained by each machine learning technique.

Model	Optimal Hyperparameters	RMSE (Test Set)		MAE (Test Set)		R^2^ Score (Test Set)	
		COF	SWR	COF	SWR	COF	SWR
**DT**	Max. Depth: **50**	**0.00214**	**5.4 × 10^−7^**	**0.0014**	**3.7 × 10^−7^**	**0.9367**	**0.9579**
**RF**	Min. Split: **3**	**0.00111**	**3.1 × 10^−7^**	**0.0007**	**2.1 × 10^−7^**	**0.9827**	**0.9861**
	Min. Leaf Samples: **1**						
	Impurity Decrease Threshold: **None**						
**SVM**	Gamma: **0.25**	**0.000209**	**0.0002**	**0.0002**	**0.00016**	**0.9999**	**0.9998**
	Kernel: **Radial Basis Function**						
	C Parameter: **100**						
	Epsilon: **0.00001**						
**KNN**	No. of Neighbours: **8**	**0.016215**	**0.01511**	**0.0105**	**0.0105**	**0.98891**	**0.99098**
	Weights based on distance						
	Distance Computation: **Euclidean**						
**ANN**	Epochs: **100**	**0.022596**	**0.03349**	**0.0184**	**0.02676**	**0.978471**	**0.95569**
	Optimizer: **“Adam”**						
	Validation amount: **20%**						
	Learning Rate: **0.001**						

## Data Availability

The dataset is available on request from the corresponding author.

## References

[1] Kulikova A.V., Diaz D.J., Chen T., Cole T.J., Ellington A.D., Wilke C.O. (2023). Two sequence- and two structure-based ML models have learned different aspects of protein biochemistry. Sci. Rep..

[2] Shahab M., Zheng G., Khan A., Wei D., Novikov A.S. (2023). Machine Learning-Based Virtual Screening and Molecular Simulation Approaches Identified Novel Potential Inhibitors for Cancer Therapy. Biomedicines.

[3] Senior A.W., Evans R., Jumper J., Kirkpatrick J., Sifre L., Green T., Qin C., Žídek A., Nelson A.W.R., Bridgland A. (2020). Improved protein structure prediction using potentials from deep learning. Nature.

[4] Chen C., Gu G. (2019). Machine learning for composite materials. MRS Commun..

[5] Butler K.T., Davies D.W., Cartwright H., Isayev O., Walsh A. (2018). Machine learning for molecular and materials science. Nature.

[6] Khanam P.N., AlMaadeed M.A., AlMaadeed S., Kunhoth S., Ouederni M., Sun D., Hamilton A., Jones E.H., Mayoral B. (2016). Optimization and Prediction of Mechanical and Thermal Properties of Graphene/LLDPE Nanocomposites by Using Artificial Neural Networks. Int. J. Polym. Sci..

[7] Vinoth A., Dey S., Datta S. (2021). Designing UHMWPE hybrid composites using machine learning and metaheuristic algorithms. Compos. Struct..

[8] Kiakojouri A., Lu Z., Mirring P., Powrie H., Wang L. (2022). A generalised machine learning model based on multinomial logistic regression and frequency features for rolling bearing fault classification, Insight-Non-Destructive Test. Cond. Monit..

[9] Nazari A., Sanjayan J.G. (2015). Modelling of compressive strength of geopolymer paste, mortar and concrete by optimized support vector machine. Ceram. Int..

[10] Zhang J., Wang Y. (2021). Evaluating the bond strength of FRP-to-concrete composite joints using metaheuristic-optimized least-squares support vector regression. Neural Comput. Appl..

[11] Hasan M.S., Kordijazi A., Rohatgi P.K., Nosonovsky M. (2022). Triboinformatics Approach for Friction and Wear Prediction of Al-Graphite Composites Using Machine Learning Methods. J. Tribol..

[12] Hasan M.S., Nosonovsky M. (2022). Triboinformatics: Machine learning algorithms and data topology methods for tribology. Surf. Innov..

[13] Singh K.S.K., Kumar S., Singh K.K. (2022). Computational data-driven based optimization of tribological performance of graphene filled glass fiber reinforced polymer composite using machine learning approach. Mater. Today Proc..

[14] Mohammed A.S., Dodla S., Katiyar J.K., Samad M.A. (2022). Prediction of friction coefficient of su-8 and its composite coatings using machine learning techniques. Proc. Inst. Mech. Eng. Part J J. Eng. Tribol..

[15] Borjali A., Monson K., Raeymaekers B. (2019). Predicting the polyethylene wear rate in pin-on-disc experiments in the context of prosthetic hip implants: Deriving a data-driven model using machine learning methods. Tribol. Int..

[16] Aliyu I.K., Azam M.U., Lawal D.U., Samad M.A. (2020). Optimization of SiC concentration and process parameters for a wear-resistant UHMWPE nancocomposite. Arab. J. Sci. Eng..

[17] Agarwal M., Singh M.K., Srivastava R., Gautam R.K. (2021). Microstructural measurement and artificial neural network analysis for adhesion of tribolayer during sliding wear of powder-chip reinforcement based composites. Measurement.

[18] Argatov I.I., Chai Y.S. (2019). An artificial neural network supported regression model for wear rate. Tribol. Int..

[19] Bhaumik S., Kamaraj M. (2021). Artificial neural network and multi-criterion decision making approach of designing a blend of biodegradable lubricants and investigating its tribological properties. Proc. Inst. Mech. Eng. Part J J. Eng. Tribol..

[20] Hasan M.S., Kordijazi A., Rohatgi P.K., Nosonovsky M. (2022). Machine learning models of the transition from solid to liquid lubricated friction and wear in aluminum-graphite composites. Tribol. Int..

[21] Kumar D.S., Rajmohan M. (2019). Optimizing Wear Behavior of Epoxy Composites Using Response Surface Methodology and Artificial Neural Networks. Polym. Compos..

[22] Liu X., Liu T., Feng P. (2022). Long-term performance prediction framework based on XGBoost decision tree for pultruded FRP composites exposed to water, humidity and alkaline solution. Compos. Struct..

[23] Costa V.G., Pedreira C.E. (2023). Recent advances in decision trees: An updated survey. Artif. Intell. Rev..

[24] Hu X., Rudin C., Seltzer M. (2019). Optimal sparse decision trees. Adv. Neural Inf. Process. Syst..

[25] Amanoul S.V., Abdulazeez A.M., Zeebare D.Q., Ahmed F.Y.H. Intrusion Detection Systems Based on Machine Learning Algorithms. Proceedings of the 2021 IEEE International Conference on Automatic Control & Intelligent Systems (I2CACIS).

[26] Borup D., Christensen B.J., Mühlbach N.S., Nielsen M.S. (2023). Targeting predictors in random forest regression. Int. J. Forecast..

[27] Chen G.H., Shah D. (2018). Explaining the success of nearest neighbor methods in prediction. Found. Trends® Mach. Learn..

[28] Borah P., Gupta D. (2021). Robust twin bounded support vector machines for outliers and imbalanced data. Appl. Intell..

[29] Awad M., Khanna R. (2015). Support vector regression. Efficient Learning Machines.

[30] Kalita D.J., Singh V.P., Kumar V. (2023). A novel adaptive optimization framework for SVM hyper-parameters tuning in non-stationary environment: A case study on intrusion detection system. Expert. Syst. Appl..

[31] Manoj I.V., Soni H., Narendranath S., Mashinini P.M., Kara F. (2022). Examination of machining parameters and prediction of cutting velocity and surface roughness using RSM and ANN using WEDM of Altemp HX. Adv. Mater. Sci. Eng..

[32] Li S., Shao M., Duan C., Yan Y., Wang Q., Wang T. (2019). Tribological behavior prediction of friction materials for ultrasonic motors using Monte Carlo-based artificial neural network. J. Appl. Polym. Sci..

